# Dietary score associations with markers of chronic low-grade inflammation: a cross-sectional comparative analysis of a middle- to older-aged population

**DOI:** 10.1007/s00394-022-02892-1

**Published:** 2022-05-05

**Authors:** Seán R. Millar, Pilar Navarro, Janas M. Harrington, Nitin Shivappa, James R. Hébert, Ivan J. Perry, Catherine M. Phillips

**Affiliations:** 1 HRB Centre for Health and Diet Research, School of Public Health, University College Cork, Cork, Ireland; 2 School of Public Health, Physiotherapy and Sports Science, University College Dublin, Dublin 4, Ireland; 3 Cancer Prevention and Control Program and Department of Epidemiology and Biostatistics, Arnold School of Public Health, University of South Carolina and Connecting Health Innovations LLC, Columbia, SC USA

**Keywords:** Diet, Scores, Chronic, Inflammation, Biomarkers

## Abstract

**Purpose:**

To assess relationships between the Dietary Approaches to Stop Hypertension (DASH), Mediterranean Diet (MD), Dietary Inflammatory Index (DII^®^) and Energy-adjusted DII (E-DII™) scores and pro-inflammatory cytokines, adipocytokines, acute-phase response proteins, coagulation factors and white blood cells.

**Methods:**

This was a cross-sectional study of 1862 men and women aged 46–73 years, randomly selected from a large primary care centre in Ireland. DASH, MD, DII and E-DII scores were derived from validated food frequency questionnaires. Correlation and multivariate-adjusted linear regression analyses with correction for multiple testing were performed to examine dietary score relationships with biomarker concentrations.

**Results:**

In fully adjusted models, higher diet quality or a less pro-inflammatory diet was associated with lower concentrations of c-reactive protein, neutrophils (all dietary scores), complement component 3 [C3], interleukin 6 [IL-6], tumour necrosis factor-alpha [TNF-α], white blood cell count [WBC], the neutrophil-to-lymphocyte ratio [NLR] (DASH, DII and E-DII), monocytes (DASH and DII) and resistin (DII and E-DII). After accounting for multiple testing, relationships with C3 (DASH: *β* = − 2.079*, p* = .011 and DII: *β* = 2.521, *p* = .036), IL-6 (DASH: *β* = − 0.063*, p* = .011), TNF-α (DASH: *β* = − 0.027*, p* = .034), WBC (DASH: *β* = − 0.028*, p* = .001 and DII: *β* = 0.029, *p* = .02), neutrophils (DASH: *β* = − 0.041*, p* = .001; DII: *β* = 0.043, *p* = .007; E-DII: *β* = 0.029, *p* = .009) and the NLR (DASH: *β* = − 0.035*, p* = .011) persisted.

**Conclusions:**

Better diet quality, determined by the DASH score, may be more closely associated with inflammatory biomarkers related to health in middle- to older-aged adults than the MD, DII and E-DII scores.

**Supplementary Information:**

The online version contains supplementary material available at 10.1007/s00394-022-02892-1.

## Introduction

Increasing evidence over the last decade has linked low-grade systemic inflammation and raised immune activation with chronic conditions including type 2 diabetes, cardiovascular disease (CVD), neurodegenerative disease and many cancers [1–6]. In addition, ageing is characterised by an increase in pro-inflammatory molecules in circulation, a phenomenon that has been termed “inflammageing” [7, 8]. Accordingly, many circulating biomarkers have been evaluated to determine disease risk and their relationships with obesity and certain lifestyle behaviours have been examined [6, 9–11].

Dietary intake may modulate inflammation and represents a promising therapeutic target to reduce metabolic dysfunction and chronic disease risk [12–15]. Consequently, numerous dietary scores have been developed with the aim of synthesising a large amount of dietary information as a single indicator useful for assessing risk factor–disease relationships [16, 17]. The Dietary Approaches to Stop Hypertension (DASH) diet emphasises consumption of fruits, vegetables, nuts, legumes, whole-grains and low-fat dairy and restricting intake of red meat, sugar, sweetened beverages, total fat and saturated fat [18]. The Mediterranean Diet (MD) is characterised by high consumption of fruits, vegetables, nuts, legumes and cereals, a low consumption of red meat and meat products, a high ratio of monounsaturated fat to saturated fat and moderate consumption of fermented dairy products and alcohol [19, 20]. More recently, the Dietary Inflammatory Index (DII^®^) and Energy-adjusted Dietary Inflammatory Index (E-DII™) were developed specifically to measure the inflammatory potential of diet based on the overall inflammatory properties of dietary components such as macronutrients, vitamins and minerals, flavonoids and other bioactive compounds [21, 22].

Although studies have indicated relationships between the DASH, MD, DII, E-DII and circulating biomarkers of inflammation [15, 23, 24], there are still questions as to which of these indices is a better marker of health outcomes, as the validity of a dietary score depends on the extent to which it is able to distinguish between individuals on relevant health-related intermediate markers [16, 25]. In addition, the focus on inflammatory profiling in this context has been restricted to a narrow range of biomarkers [26]. Therefore, the aim of the present study was to assess cross-sectional relationships between the DASH, MD, DII and E-DII scores and a range of pro-inflammatory cytokines, adipocytokines, acute-phase response proteins, coagulation factors and white blood cells, using a random sample of 1862 middle- to older-aged men and women, to determine which dietary score is more closely associated with biomarkers of systemic low-grade inflammation.

## Materials and methods

### Study population and setting

The Cork and Kerry Diabetes and Heart Disease Study (Phase II—Mitchelstown Cohort) was a single-centre study conducted between 2010 and 2011. A random sample was recruited from a large primary care centre in Mitchelstown, County Cork, Ireland. The Living Health Clinic serves a population of approximately 20,000 predominantly White European subjects, with a mix of urban and rural residents. Stratified sampling was employed to recruit equal numbers of men and women from all registered attending patients in the 45–70-year age group. In total, 3807 potential participants were selected from the practice list. Following the exclusion of duplicates, deaths and subjects incapable of consenting or attending appointment, 3051 were invited to participate in the study and of these, about two-thirds (2047, 49% male) completed the questionnaire and physical examination components of the baseline assessment. Dietary data were available for 1862 subjects. Details regarding the study design, sampling procedures and methods of data collection have been reported previously [27]. Ethics committee approval conforming to the Declaration of Helsinki was obtained from the Clinical Research Ethics Committee of University College Cork. A letter signed by the contact GP in the clinic was sent out to all selected participants with a reply slip indicating acceptance or refusal. All participants gave signed informed consent, including permission to use their data for research purposes.

### Clinical procedures

Study participants attended the clinic in the morning after an overnight fast and blood samples were taken on arrival. Fasting glucose and glycated haemoglobin A_1c_ (HbA_1c_) concentrations were measured in fresh samples by Cork University Hospital Biochemistry Laboratory using standardised procedures. Glucose concentrations were determined using a glucose hexokinase assay (Olympus Life and Material Science Europa Ltd., Lismeehan, Co. Clare, Ireland) and HbA_1c_ levels were measured in the haematology laboratory on an automated high-pressure liquid chromatography instrument Tosoh G7 [Tosoh HLC-723 (G7), Tosoh Europe N.V, Tessenderlo, Belgium]. C-reactive protein (CRP), tumour necrosis factor-alpha (TNF-α), interleukin 6 (IL-6), adiponectin, leptin, resistin and plasminogen activator inhibitor-1 (PAI-1) were assessed using a biochip array system (Evidence Investigator; Randox Laboratories, UK). Complement component 3 (C3) was measured by immunoturbidimetric assay (RX Daytona; Randox Laboratories). White blood cell count (WBC), neutrophil, lymphocyte, monocyte, eosinophil and basophil concentrations were determined by flow cytometry technology as part of a full blood count. The neutrophil-to-lymphocyte ratio (NLR) was calculated as neutrophils divided by lymphocytes.

Anthropometric measurements were performed by trained researchers with reference to a standard operating procedures manual. Height was measured with a portable Seca Leicester height/length stadiometer (Seca, Birmingham, UK) and weight was measured using a portable electronic Tanita WB-100MA weighing scale (Tanita Corp, IL, USA). The weighing scale was placed on a firm flat surface and was calibrated weekly. Body mass index (BMI) was calculated as weight in kilograms divided by the square of height in meters.

### Data collection

A general health and lifestyle questionnaire assessed demographic variables, lifestyle behaviours and morbidity. Information on sex, age, education, prescription anti-inflammatory medication use, smoking status, diagnosis of type 2 diabetes and cancer was provided by participants. The presence of CVD was obtained by asking study participants if they had been diagnosed with any one of the following seven conditions: Heart Attack (including coronary thrombosis or myocardial infarction), Heart Failure, Angina, Aortic Aneurysm, Hardening of the Arteries, Stroke or any other Heart Trouble. Subjects who indicated a diagnosis of any one of these conditions were classified as having CVD. Physical activity levels were measured using the validated International Physical Activity Questionnaire (IPAQ) [28].

### Dietary assessment

A Food Frequency Questionnaire (FFQ) was used for dietary assessment. Diet was evaluated using a modified version of the self-completed European Prospective Investigation into Cancer and Nutrition (EPIC) FFQ [29], which has been validated extensively in several populations [30]. Adapted to reflect the Irish diet, the 150-item semi-quantitative FFQ used in the current study was originally validated for use in the Irish population using food diaries and a protein biomarker in a volunteer sample [31] and incorporated into the SLÁN Irish National Surveys of Lifestyle Attitudes and Nutrition 1998, 2002 and 2007 [32–34]. The FFQ was also validated using a 7-day weighed food record completed in another Irish study (Lifeways Cross-generational Study), with reasonable agreement for fat, carbohydrate, and their components, and with lower agreement for protein [35]. The average medium serving of each food item consumed by participants over the last 12 months was converted into quantities using standard portion sizes. Food item quantity was expressed as (g/d) and beverages as (ml/d). The daily intake of energy and nutrients was computed from FFQ data using a tailored computer programme (FFQ Software Version 1.0; developed by the National Nutrition Surveillance Centre, School of Public Health, Physiotherapy and Sports Science, University College Dublin, Belfield, Dublin 4, Ireland), which linked frequency selections with the food equivalents in McCance and Widdowson Food Tables [36].

### DASH score

Based on the FFQ, the DASH score was constructed. DASH is a dietary pattern rich in fruits, vegetables, whole grains and low-fat dairy foods and is limited in sugar-sweetened foods and beverages, red meat and added fats. This diet has been promoted by the National Heart, Lung and Blood Institute (part of the National Institutes of Health, a United States government organisation) to prevent and control hypertension. For each food group, consumption was divided into quintiles, and participants were classified according to their intake ranking. Consumption of healthy food components were rated on a scale of 1–5; the higher the score, the more frequent the consumption of that food (i.e., those in quintile 1 had the lowest consumption and received a score of 1; conversely, those in quintile 5 had the highest consumption and received a score of 5). Less-healthy dietary constituents, where low consumption is desired, were scored on a reverse scale, with lower consumption receiving higher scores. Component scores were summed, and an overall DASH score was calculated for each person. DASH diet scores ranged from 11 to 42. Lower scores represent poorer and higher scores represent better quality diet [37].

### MD score

A scale indicating the degree of adherence to the traditional Mediterranean diet was developed by Trichopoulou et al. [38] and revised to include fish intake [19]. This score is proposed for implementation and uptake in non-Mediterranean countries such as Ireland for incorporation into current Irish dietary guidelines [20, 39]. Scoring is based on intake of 9 items: vegetables, legumes, fruit and nuts, dairy products, cereals, meat and meat products, fish, alcohol and the ratio of monounsaturated to saturated fat. A value of 0 or 1 was assigned to each of nine items with use of the sex-specific median as the cut-off. For beneficial components (vegetables, legumes, fruits and nuts, cereal and fish), consumption above the study median received 1 point; all other intakes received 0 points. For components presumed to be detrimental (dairy products, meat and meat products), consumption below the median received 1 point. For fat intake, we used the ratio of monounsaturated lipids to saturated lipids. For ethanol, men who consumed 10–50 g/day and women who consumed 5–25 g/day received 1 point; otherwise, the score was 0. Thus, the total MD score ranged from 0 (minimal adherence to the traditional Mediterranean diet) to 9 (maximal adherence).

### DII and E-DII scores

DII scores were calculated using a method previously reported by Shivappa et al. [22]. Briefly, the scoring algorithm based on an extensive review of the literature focused on the effect of diet on six inflammatory biomarkers (IL-1β, IL-4, IL-6, IL-10, TNF-α and CRP) from 1950 to 2010. A total of 26 of the 45 possible food parameters were used for DII calculation based on the FFQ in this study and these were as follows: carbohydrate, protein, fat, alcohol, fibre, cholesterol, saturated fat, monounsaturated fat, polyunsaturated fat, niacin, thiamine, riboflavin, vitamin B12, vitamin B6, iron, magnesium, zinc, selenium, vitamin A, vitamin C, vitamin D, vitamin E, folic acid, onion, garlic and tea. Dietary information for each study participant was linked to a regionally representative database that provides a global estimate of mean intake for each of the foods, nutrients and other food components along with its standard deviation considered in the DII definition [40]. These parameters were then used to derive the participant’s exposure relative to the standard global mean as a z-score, derived by subtracting the mean of the regionally representative database from the amount reported and dividing this value by the parameter’s standard deviation. These *z*-scores were then converted to proportions (i.e., with values ranging from 0 to 1) and then centred by doubling and subtracting 1. The resulting value was then multiplied by the corresponding food parameter effect score (derived from a literature review on the basis of 1943 peer-reviewed articles) [40]. All of the food parameter-specific DII scores were then summed to create the overall DII score for every participant in the study. The E-DII scores were calculated by converting raw dietary components to amount per 1000 kcal, and then repeating a process analogous to that used for the DII but employing an energy-adjusted global comparison database [41]. For both the DII and E-DII, higher scores are more pro-inflammatory and lower scores are anti-inflammatory.

### Classification and scoring of variables

Categories of education included ‘some primary (not complete)’, ‘primary or equivalent’, ‘intermediate/group certificate or equivalent’, ‘leaving certificate or equivalent’, ‘diploma/certificate’, ‘primary university degree’ and ‘postgraduate/higher degree’. These were collapsed and recoded into a dichotomous variable: ‘primary education only’ (finished full-time education at 13 years or younger) and ‘intermediate or higher’. Type 2 diabetes was determined as a fasting glucose level ≥ 7.0 mmol/l or a HbA_1c_ level ≥ 6.5% (≥ 48 mmol/mol) [42] or by self-reported diagnosis.

Smoking status was defined as follows: (i) never smoked, i.e., having never smoked at least 100 cigarettes (5 packs) in their entire life; (ii) former smoker, i.e., having smoked 100 cigarettes in their entire life and do not smoke at present; and (iii) current smoker, i.e., smoking at present. These definitions were the same as those used in the SLÁN National Health and Lifestyle Survey [43]. Physical activity was categorised as low, moderate and high levels of activity using the IPAQ. This was then recoded as a dichotomous variable: ‘moderate/high’ or ‘low’ physical activity.

### Statistical analysis

Descriptive characteristics were examined according to sex and dietary score quartiles. Categorical features are presented as percentages and continuous variables are shown as a mean (plus or minus one standard deviation) or a median and interquartile range for skewed data. Differences were analysed using a Pearson’s chi-square test, Student’s *t*-test or a Mann Whitney *U*. Trend relationships were determined using a Jonckheere test, a linear-by-linear chi-square or an ANOVA. Correlations between dietary scores and dietary markers were examined using Spearman’s rank-order correlation. Partial non-parametric tests examined correlations between dietary indices and inflammatory and thrombotic biomarkers. Dietary scores were standardised and skewed biomarker data were log-transformed. Linear regression analysis was performed to determine DASH, MD, DII and E-DII score associations with biomarker concentrations. Two models were run: the first model was adjusted for sex and age; a second model was adjusted for sex, age, education, use of anti-inflammatory medications, type 2 diabetes, CVD, cancer, smoking status, physical activity, BMI and total energy intake. Models which examined the E-DII were not adjusted for total energy intake as this was accounted for in the formulation of the E-DII score. To correct for the multiple testing performed, we calculated false discovery rate (FDR) adjusted *p* values via the Romano-Wolf multiple hypothesis correction method using the **rwolf** command in Stata [44]. Data analysis was conducted using Stata SE Version 13 (Stata Corporation, College Station, TX, USA) for Windows. Partial Spearman correlation coefficients were determined using IBM SPSS Statistics 25 (IBM Corp., Armonk, NY, USA) [45]. For all analyses, a *p* value (two-tailed) of less than 0.05 was considered to indicate statistical significance.

## Results

### Descriptive characteristics

Characteristics of the study population for the full sample and according to sex are presented in Table 1. Significant differences between the sexes were noted for education, use of anti-inflammatory medications, type 2 diabetes, CVD, cancer, physical activity, BMI and each dietary score. Sex differences also were observed for all biomarker levels except for lymphocyte and basophil concentrations.

**Table 1:** Characteristics of the study population – full sample and stratified by sex

Variable	Full sample	Males	Females	*p*
	*n* = 1862	*n* = 911	*n* = 951	
Age (median)	59.0 (54.0–63.0)	59.0 (54.0–63.0)	59.0 (54.0–64.0)	.927
Primary education only (%)	461 (26.2)	264 (30.2)	197 (22.2)	< .002
On anti-inflammatory medications (%)	299 (16.3)	186 (20.9)	113 (12.0)	< .001
Type 2 diabetes (%)	159 (8.5)	100 (11.0)	59 (6.2)	< .001
CVD (%)	193 (10.4)	130 (14.3)	63 (6.6)	< .001
Cancer (%)	72 (3.9)	23 (2.5)	49 (5.2)	.003
Never smoker (%)	957 (51.9)	399 (44.1)	558 (59.3)	< .001
Former smoker (%)	623 (33.8)	374 (41.4)	249 (26.5)	
Current smoker (%)	265 (14.4)	131 (14.5)	134 (14.2)	
Low-level physical activity (%)	841 (47.2)	352 (41.2)	489 (52.9)	< .001
BMI, kg/m^2^ (mean)	28.4 ± 4.6	29.0 ± 4.0	27.9 ± 5.0	< .001
Energy intake, kcal (mean)	2059.1 ± 797.6	2082.5 ± 789.4	2036.8 ± 805.2	.217
DASH score (mean)	26.8 ± 5.4	25.0 ± 5.0	28.5 ± 5.1	< .001
MD score (mean)	4.23 ± 1.9	4.40 ± 1.9	4.06 ± 1.8	< .001
DII score (mean)	− 0.32 ± 1.6	− 0.08 ± 1.6	− 0.54 ± 1.5	< .001
E-DII score (mean)	− 0.78 ± 1.4	− 0.43 ± 1.4	− 1.11 ± 1.3	< .001
C3, mg/dl (mean)	135.62 ± 24.6	133.67 ± 22.4	137.48 ± 26.5	.001
CRP, ng/ml (median)	1.34 (0.97–2.28)	1.32 (0.95–2.11)	1.37 (0.99–2.45)	.035
IL-6, pg/ml (median)	1.77 (1.19–2.84)	1.90 (1.25–3.02)	1.67 (1.13–2.67)	< .001
TNF-α, pg/ml (median)	5.95 (4.88–7.28)	6.06 (5.07–7.47)	5.84 (4.71–7.10)	< .001
Adiponectin, ng/ml (median)	4.78 (2.93–7.55)	3.32 (2.22–4.97)	6.69 (4.47–9.61)	< .001
Leptin, ng/ml (median)	1.91 (1.08–3.04)	1.55 (0.77–2.53)	2.25 (1.26–4.22)	< .001
Resistin, ng/ml (median)	5.03 (3.91–6.67)	4.87 (3.81–6.50)	5.20 (3.99–6.94)	.007
PAI-1, ng/ml (mean)	27.32 ± 12.5	28.94 ± 12.8	25.79 ± 11.9	< .001
WBC, 10^9^/l (median)	5.70 (4.80–6.80)	5.90 (5.00–7.00)	5.50 (4.60–6.50)	< .001
Neutrophils, 10^9^/l (median)	3.11 (2.51–3.92)	3.25 (2.65–4.13)	2.97 (2.37–3.76)	< .001
Lymphocytes, 10^9^/l (median)	1.74 (1.42–2.14)	1.73 (1.41–2.14)	1.76 (1.44–2.15)	.321
NLR (median)	1.77 (1.39–2.28)	1.86 (1.49–2.38)	1.67 (1.31–2.17)	< .001
Monocytes, 10^9^/l (median)	0.50 (0.40–0.62)	0.54 (0.44–0.68)	0.45 (0.36–0.56)	< .001
Eosinophils, 10^9^/l (median)	0.17 (0.11–0.26)	0.19 (0.12–0.28)	0.15 (0.10–0.24)	< .001
Basophils, 10^9^/l (median)	0.03 (0.02–0.04)	0.03 (0.02–0.04)	0.03 (0.02–0.04)	.512

Mean ± one standard deviation, median (interquartile range) and numbers (percentages) are shown. *p*-values determined from a Mann Whitney U, Student’s *t*-test or a Pearson’s chi-square

*C3* complement component 3, *CRP* c-reactive protein, *CVD* cardiovascular disease, *DASH* Dietary Approaches to Stop Hypertension, *DII* Dietary Inflammatory Index, *E-DII* Energy-adjusted Dietary Inflammatory Index, *IL-6* interleukin 6, *MD* Mediterranean Diet, *TNF-α* tumour necrosis factor-alpha, *PAI-1* plasminogen activator inhibitor 1, *WBC* white blood cell count, *NLR* neutrophil-to-lymphocyte ratio

In Supplementary Table S1, characteristics of the study population were examined according to dietary score quartiles. In general, subjects who had poorer dietary quality or the most pro-inflammatory diets were more likely to be male, have lower educational levels and type 2 diabetes, were more likely to be current smokers, reported lower physical activity levels and had higher (lower for adiponectin) concentrations of inflammatory and thrombotic biomarkers than did those who consumed higher quality/less pro-inflammatory diets.

### Correlation analysis

In correlation analyses which examined relationships between dietary scores and dietary markers (Table 2), better diet quality or a less inflammatory diet was correlated with reduced consumption of saturated fatty acids and red meat (DASH, MD, E-DII) and greater consumption of polyunsaturated fatty acids (MD and DII). Higher correlations between the DII and micronutrients were observed when compared to other dietary indices. For all scores, better quality or more anti-inflammatory diets were correlated with higher consumption of white fish, oily fish and shellfish, fruits, vegetables and legumes, fibre, whole grains, nuts, onions and garlic. Negative and positive correlations with sweet snack products were strong for the DASH and E-DII indices respectively, when compared to the MD and DII scores. In addition, examination of daily food pyramid shelf servings demonstrated that poorer quality/more inflammatory diets indicated by the DASH and E-DII scores were more highly correlated with consumption of high fat/sugar foods and beverages than the MD and DII scores, which showed weak correlations.

**Table 2:** Spearman correlation coefficients between dietary scores and dietary markers

Dietary marker	DASH score		MD score		DII score		E-DII score	
	*ρ* coefficient	*p*	*ρ* coefficient	*p*	*ρ* coefficient	*p*	*ρ* coefficient	*p*
**Macronutrients**								
Fat, g/d	− 0.246	** < .001**	− 0.073	**.002**	− 0.504	** < .001**	0.406	** < .001**
SFA, g/d	− 0.326	** < .001**	− 0.242	** < .001**	− 0.331	** < .001**	0.561	** < .001**
PUFA, g/d	− 0.093	** < .001**	0.115	** < .001**	− 0.602	** < .001**	0.130	** < .001**
MUFA, g/d	− 0.262	** < .001**	− 0.046	**.048**	− 0.493	** < .001**	0.386	** < .001**
Carbohydrate, g/d	− 0.015	.523	0.153	** < .001**	− 0.705	** < .001**	0.117	** < .001**
Protein, g/d	− 0.071	**.002**	0.121	** < .001**	− 0.702	** < .001**	0.056	**.016**
Sugar, g/d	0.153	** < .001**	0.219	** < .001**	− 0.656	** < .001**	− 0.003	.889
Fibre, g/d	0.309	** < .001**	0.411	** < .001**	− 0.872	** < .001**	− 0.238	** < .001**
Alcohol, ml/d	− 0.054	**.019**	0.088	** < .001**	− 0.089	** < .001**	− 0.039	.09
Cholesterol, mg/d	− 0.286	** < .001**	− 0.122	** < .001**	− 0.391	** < .001**	0.333	** < .001**
**Micronutrients**								
Sodium, mg/d	− 0.181	** < .001**	0.084	** < .001**	− 0.597	** < .001**	0.178	** < .001**
Niacin, mg/d	− 0.016	.493	0.222	** < .001**	− 0.721	** < .001**	− 0.076	**.001**
Thiamine, mg/d	0.030	.19	0.174	** < .001**	− 0.782	** < .001**	− 0.061	**.009**
Riboflavin, mg/d	0.046	**.047**	0.136	** < .001**	− 0.704	** < .001**	− 0.015	.51
Vitamin B12, μg/d	− 0.047	**.041**	0.097	** < .001**	− 0.481	** < .001**	− 0.041	.076
Vitamin B6, mg/d	0.089	** < .001**	0.245	** < .001**	− 0.813	** < .001**	− 0.154	** < .001**
Iron, mg/d	0.055	**.017**	0.240	** < .001**	− 0.794	** < .001**	− 0.067	**.004**
Magnesium, mg/d	0.224	** < .001**	0.349	** < .001**	− 0.862	** < .001**	− 0.163	** < .001**
Zinc, mg/d	− 0.087	** < .001**	0.073	**.002**	− 0.662	** < .001**	0.054	**.02**
Selenium, μg/d	− 0.119	** < .001**	0.067	**.004**	− 0.562	** < .001**	0.102	** < .001**
Retinol, μg/d	− 0.192	** < .001**	− 0.163	** < .001**	− 0.269	** < .001**	0.309	** < .001**
Carotene, μg/d	0.329	** < .001**	0.305	** < .001**	− 0.635	** < .001**	− 0.303	** < .001**
Vitamin C, mg/d	0.474	** < .001**	0.407	** < .001**	− 0.765	** < .001**	− 0.494	** < .001**
Vitamin D, μg/d	− 0.145	** < .001**	0.061	**.009**	− 0.472	** < .001**	0.114	** < .001**
Vitamin E, mg/d	0.183	** < .001**	0.282	** < .001**	− 0.707	** < .001**	− 0.175	** < .001**
Folic acid, μg/d	0.230	** < .001**	0.316	** < .001**	− 0.868	** < .001**	− 0.250	** < .001**
**Food items**								
Red meat, g/d	− 0.476	** < .001**	− 0.248	** < .001**	− 0.205	** < .001**	0.274	** < .001**
Fried fish, g/d	− 0.185	** < .001**	0.007	.751	0.002	.937	0.220	** < .001**
White fish, g/d	0.192	** < .001**	0.262	** < .001**	− 0.283	** < .001**	− 0.209	** < .001**
Oily fish, g/d	0.268	** < .001**	0.338	** < .001**	− 0.394	** < .001**	− 0.321	** < .001**
Shellfish, g/d	0.133	** < .001**	0.154	** < .001**	− 0.165	** < .001**	− 0.148	** < .001**
Fruits, g/d	0.588	** < .001**	0.415	** < .001**	− 0.586	** < .001**	− 0.431	** < .001**
Vegetables, g/d	0.455	** < .001**	0.435	** < .001**	− 0.701	** < .001**	− 0.481	** < .001**
Legumes, g/d	0.270	** < .001**	0.524	** < .001**	− 0.415	** < .001**	− 0.178	** < .001**
Whole grains, g/d	0.287	** < .001**	0.294	** < .001**	− 0.426	** < .001**	− 0.185	** < .001**
Nuts, g/d	0.184	** < .001**	0.239	** < .001**	− 0.230	** < .001**	− 0.083	** < .001**
Onions, g/d	0.161	** < .001**	0.201	** < .001**	− 0.348	** < .001**	− 0.188	** < .001**
Garlic, g/d	0.270	** < .001**	0.241	** < .001**	− 0.276	** < .001**	− 0.264	** < .001**
Tea, g/d	− 0.060	**.011**	− 0.051	**.029**	− 0.091	** < .001**	0.108	** < .001**
Low fat dairy, g/d	0.493	** < .001**	0.170	** < .001**	− 0.336	** < .001**	− 0.244	** < .001**
Sweet snacks, g/d	− 0.487	** < .001**	− 0.138	** < .001**	− 0.076	**.001**	0.469	** < .001**
Salty snacks, g/d	− 0.427	** < .001**	− 0.032	.171	− 0.199	** < .001**	0.183	** < .001**
**Food pyramid shelf servings**^a^								
Bread, cereal, potatoes, grains and rice	− 0.039	.09	0.077	**.001**	− 0.491	** < .001**	0.097	** < .001**
Fruit and vegetables	0.580	** < .001**	0.511	** < .001**	− 0.780	** < .001**	− 0.526	** < .001**
Dairy	− 0.001	.968	− 0.007	.752	− 0.197	** < .001**	0.124	** < .001**
Meat, fish, poultry and eggs	− 0.270	** < .001**	− 0.016	.485	− 0.401	** < .001**	0.133	** < .001**
High fat/sugar foods/drinks	− 0.410	** < .001**	− 0.165	** < .001**	− 0.215	** < .001**	0.516	** < .001**

*p* value of < 0.05 was considered to be statistically significant. *p* values are highlighted in bold

Values are presented as Spearman correlation coefficients between continuous dietary scores and dietary markers among the Mitchelstown Cohort (n = 1862). Significant *p* **highlighted. **For the DASH and MD, lower scores represent poorer and higher scores represent better quality diet. For the DII and E-DII, higher scores are more pro-inflammatory and lower scores are anti-inflammatory

*DII* DASH: Dietary Approaches to Stop Hypertension, Dietary Inflammatory Index, *E-DII* Energy-adjusted Dietary Inflammatory Index, *MD* Mediterranean Diet, *MUFA* monounsaturated fatty acids, *PUFA* polyunsaturated fatty acids, *SFA* saturated fatty acids

^a^Daily number of servings based on Irish food pyramid recommendations [39]

Partial Spearman correlation coefficients between dietary scores and inflammatory and thrombotic biomarkers are shown in Table 3. Better dietary quality (a higher DASH or MD score) was significantly negatively correlated with C3 (DASH only), CRP, IL-6, TNF-α, WBC, neutrophil (DASH and MD), NLR and monocyte (DASH only) concentrations. A more pro-inflammatory diet indicated by higher DII or E-DII scores was significantly positively correlated with C3, CRP, IL-6, TNF-α, resistin, WBC, neutrophils, the NLR (DII and E-DII) and monocytes (DII only). No significant relationships were observed between dietary scores and adiponectin, leptin, PAI-1, lymphocyte, eosinophil or basophil levels. As descriptive analyses suggested current smoking to be correlated with biomarker levels, we conducted sensitivity analyses which excluded current smokers (Supplementary Table S2). Similar correlations between dietary scores and biomarkers were observed. However, relationships between dietary indices and concentrations of C3 (E-DII), IL-6 (MD), resistin (DII and E-DII), WBC (MD and E-DII), neutrophils (MD) and monocytes (DII) were non-significant.

**Table 3:** Partial Spearman correlation coefficients between dietary scores and inflammatory and thrombotic biomarkers

Biomarker	DASH score		MD score		DII score		E-DII score	
	*ρ* coefficient	*p*	*ρ* coefficient	*p*	*ρ* coefficient	*p*	*ρ* coefficient	*p*
C3, mg/dl	− 0.094	** < .001**	− 0.040	.114	0.070	**.005**	0.056	**.026**
CRP, ng/ml	− 0.059	**.018**	− 0.073	**.004**	0.077	**.002**	0.081	**.001**
IL-6, pg/ml	− 0.089	** < .001**	− 0.060	**.016**	0.082	**.001**	0.095	** < .001**
TNF-α, pg/ml	− 0.097	** < .001**	− 0.053	**.035**	0.076	**.002**	0.099	** < .001**
Adiponectin, ng/ml	0.029	.245	− 0.019	.45	− 0.013	.594	− 0.001	.96
Leptin, ng/ml	− 0.023	.364	− 0.027	.278	0.044	.08	0.042	.094
Resistin, ng/ml	− 0.025	.323	0.004	.883	0.062	**.013**	0.057	**.023**
PAI-1, ng/ml	− 0.030	.236	0.025	.327	0.009	.726	− 0.007	.777
WBC, 10^9^/l	− 0.104	** < .001**	− 0.060	**.016**	0.102	** < .001**	0.072	**.004**
Neutrophils, 10^9^/l	− 0.118	** < .001**	− 0.077	**.002**	0.125	** < .001**	0.096	** < .001**
Lymphocytes, 10^9^/l	− 0.022	.383	− 0.014	.588	0.007	.792	0.002	.926
NLR	− 0.074	**.003**	− 0.040	.111	0.091	** < .001**	0.072	**.004**
Monocytes, 10^9^/l	− 0.077	**.002**	− 0.048	.053	0.054	**.029**	0.033	.187
Eosinophils, 10^9^/l	− 0.032	.194	− 0.010	.686	0.017	.498	0.005	.84
Basophils, 10^9^/l	− 0.022	.388	0.001	.981	0.011	.645	0.018	.479

*p* value of < 0.05 was considered to be statistically significant. *p* values are highlighted in bold

Models adjusted for sex, age, education, use of anti-inflammatory medications, type 2 diabetes, CVD, cancer, never/former/current smoker, physical activity, BMI and total energy intake. Models which examine the E-DII score do not adjust for total energy intake. Values are presented as partial Spearman correlation coefficients between continuous dietary scores and inflammatory and thrombotic biomarkers among the Mitchelstown Cohort (n = 1862). Significant *p* **highlighted. **For the DASH and MD, lower scores represent poorer and higher scores represent better quality diet. For the DII and E-DII, higher scores are more pro-inflammatory and lower scores are anti-inflammatory

*C3* complement component 3, *CRP* c-reactive protein, *DASH* Dietary Approaches to Stop Hypertension, *DII* Dietary Inflammatory Index, *E-DII* Energy-adjusted Dietary Inflammatory Index, *IL-6* interleukin 6, *MD* Mediterranean Diet, *TNF-α* tumour necrosis factor-alpha, *PAI-1* plasminogen activator inhibitor 1, *WBC* white blood cell count, *NLR* neutrophil-to-lymphocyte ratio

### Linear regression

Linear regression analyses describing associations between dietary scores and inflammatory biomarkers are shown in Table 4. In fully adjusted models, higher diet quality or a less pro-inflammatory diet was associated with lower concentrations of CRP, neutrophils (all dietary scores), C3, IL-6, TNF-α, WBC, the NLR (DASH, DII and E-DII), monocytes (DASH and DII) and resistin (DII and E-DII). After accounting for multiple testing, relationships with C3 (DASH: *β* = − 2.079*, p* = 0.011 and DII: *β* = 2.521, *p* = 0.036), IL-6 (DASH: *β* = − 0.063*, p* = 0.011), TNF-α (DASH: *β* = − 0.027*, p* = 0.034), WBC (DASH: *β* = − 0.028*, p* = 0.001 and DII: *β* = 0.029, *p* = 0.02), neutrophils (DASH: *β* = − 0.041*, p* = 0.001; DII: *β* = 0.043, *p* = 0.007; E-DII: *β* = 0.029, *p* = 0.009) and the NLR (DASH: *β* = − 0.035*, p* = 0.011) persisted.

**Table 4:** Linear regression analysis of the associations between dietary scores and inflammatory and thrombotic biomarkers (n = 1862)

Biomarker	DASH score			MD score			DII score			E-DII score		
	*β*	*p*	*p* (FDR)	*β*	*p*	*p* (FDR)	*β*	*p*	*p* (FDR)	*β*	*p*	*p* (FDR)
**C3**												
Model 1	− 2.201	** < .001**	**.003**	− 1.313	**.024**	.225	1.346	**.022**	.132	1.462	**.014**	.116
Model 2	− 2.079	**.001**	**.011**	− 0.942	.103	.68	2.521	**.002**	**.036**	1.273	**.032**	.24
**Log CRP**												
Model 1	− 0.062	** < .001**	**.003**	− 0.048	**.004**	**.046**	0.046	**.006**	.076	0.047	**.005**	.09
Model 2	− 0.045	**.011**	.074	− 0.040	**.014**	.174	0.063	**.005**	.076	0.041	**.014**	.153
**Log IL-6**												
Model 1	− 0.076	** < .001**	**.001**	− 0.043	**.013**	.128	0.043	**.013**	.109	0.056	**.001**	**.023**
Model 2	− 0.063	**.001**	**.011**	− 0.034	.05	.455	0.060	**.013**	.151	0.052	**.004**	.058
**Log TNF-α**												
Model 1	− 0.031	** < .001**	**.003**	− 0.012	.152	.616	0.007	.416	.868	0.023	**.006**	.096
Model 2	− 0.027	**.004**	**.034**	− 0.012	.183	.831	0.025	**.038**	.298	0.022	**.016**	.161
**Log adiponectin**												
Model 1	0.010	.492	.543	0.000	.975	.985	− 0.003	.83	.976	− 0.010	.482	.862
Model 2	0.014	.387	.762	− 0.005	.737	.994	− 0.008	.683	.961	− 0.004	.815	.985
**Log leptin**												
Model 1	− 0.020	.356	.543	− 0.033	.114	.616	0.018	.38	.868	0.022	.301	.77
Model 2	− 0.029	.155	.669	− 0.021	.263	.877	0.024	.347	.855	0.023	.236	.804
**Log resistin**												
Model 1	− 0.019	.076	.298	− 0.003	.759	.982	0.027	**.009**	.079	0.028	**.007**	.096
Model 2	− 0.012	.304	.762	0.002	.834	.994	0.038	**.009**	.119	0.027	**.012**	.153
**PAI-1**												
Model 1	− 0.635	**.04**	.182	− 0.036	.903	.985	0.033	.911	.976	− 0.028	.488	.862
Model 2	− 0.396	.239	.762	0.208	.501	.98	− 0.631	.141	.639	− 0.374	.239	.804
**Log WBC**												
Model 1	− 0.040	** < .001**	**.001**	− 0.021	**.001**	**.018**	0.033	** < .001**	**.001**	0.025	** < .001**	**.005**
Model 2	− 0.028	** < .001**	**.001**	− 0.011	.091	.653	0.029	**.001**	**.02**	0.018	**.007**	.098
**Log neutrophils**												
Model 1	− 0.052	** < .001**	**.001**	− 0.028	**.001**	**.01**	0.043	** < .001**	**.001**	0.036	** < .001**	**.001**
Model 2	− 0.041	** < .001**	**.001**	− 0.018	**.028**	.293	0.043	** < .001**	**.007**	0.029	** < .001**	**.009**
**Log lymphocytes**												
Model 1	− 0.021	**.01**	.059	− 0.013	.1	.616	0.018	**.017**	.132	0.011	.152	.641
Model 2	− 0.006	.483	.762	− 0.004	.574	.984	0.011	.333	.855	0.002	.77	.985
**Log NLR**												
Model 1	− 0.031	**.001**	**.005**	− 0.015	.107	.616	0.025	**.008**	.078	0.025	**.009**	.096
Model 2	− 0.035	**.001**	**.011**	− 0.013	.186	.831	0.032	**.018**	.183	0.027	**.008**	.104
**Log monocytes**												
Model 1	− 0.034	** < .001**	**.001**	− 0.023	**.002**	**.025**	0.035	** < .001**	**.001**	0.020	**.007**	.096
Model 2	− 0.020	**.014**	.083	− 0.012	.111	.68	0.024	**.024**	.216	0.012	.139	.643
**Log eosinophils**												
Model 1	− 0.021	.177	.431	− 0.010	.5	.956	0.026	.076	.363	0.011	.469	.862
Model 2	− 0.019	.262	.762	− 0.003	.838	.994	0.006	.766	.961	0.003	.865	.985
**Log basophils**												
Model 1	− 0.020	.145	.431	− 0.009	.505	.956	0.016	.222	.699	0.016	.237	.744
Model 2	− 0.015	.328	.762	− 0.001	.929	.994	0.002	.919	.961	0.009	.543	.954

*p* value of < 0.05 was considered to be statistically significant. *p* values are highlighted in bold

Model 1: adjusted for sex and age. Model 2: adjusted for sex, age, education, use of anti-inflammatory medications, type 2 diabetes, CVD, cancer, never/former/current smoker, physical activity, BMI and total energy intake. Models which examine the E-DII score do not adjust for total energy intake. Unstandardised β coefficients are shown. Significant *p* **highlighted. **For the DASH and MD, lower scores represent poorer and higher scores represent better quality diet. For the DII and E-DII, higher scores are more pro-inflammatory and lower scores are anti-inflammatory

*C3* complement component 3, *CRP* c-reactive protein, *DASH* Dietary Approaches to Stop Hypertension, *DII* Dietary Inflammatory Index, *E-DII* Energy-adjusted Dietary Inflammatory Index, *FDR* false discovery rate, *IL-6* interleukin 6, *MD* Mediterranean Diet, *TNF-α* tumour necrosis factor-alpha, *PAI-1* plasminogen activator inhibitor 1, *WBC* white blood cell count, *NLR* neutrophil-to-lymphocyte ratio

## Discussion

In this study of 1862 middle- to older-aged men and women we compared 4 dietary score relationships with markers of chronic low-grade inflammation. We report significant associations between dietary quality and concentrations of C3, CRP, IL-6, TNF-α, resistin, WBC, neutrophils, the NLR and monocytes in analyses which adjusted for a range of potential confounders. Consistent with previous research, these results suggest reduced systemic inflammation as a potential biological mechanism linking a higher quality healthy diet with beneficial health effects [15, 17]. The DASH score demonstrated the greatest number of significant relationships with markers of low-grade inflammation and raised immune activation in models which accounted for multiple testing. Collectively, these findings provide evidence that better diet quality, determined by the DASH score, may be more closely associated with inflammatory biomarkers related to health in middle- to older-aged adults than the MD, DII and E-DII scores.

Chronic low-grade inflammation is a major contributor to chronic conditions including metabolic syndrome, type 2 diabetes and CVD [24]. Low-grade systemic inflammation may also promote development of cancer and CVD by increasing levels of reactive oxygen and nitrogen (“free radicals”), which can damage DNA and affect endothelial function. In addition, inflammatory cytokines are thought to activate transcription factors that promote cancer progression through changes in signalling pathways that promote cell proliferation and resistance to cell death [46]. The origins of low-grade inflammation are multifactorial. Although the production of inflammatory mediators is an essential mechanism by which leukocytes confer immune protection in response to tissue injury, excessive weight gain leads to adipose tissue remodelling with the release of adipose-derived pro-inflammatory cytokines and free fatty acids into circulation, leading to metabolic dysfunction [47] and increased risk of chronic disease [15, 48].

Evidence suggests that some foods, food components and nutrients modulate inflammatory status [24]. Nutrients such as vitamins C and E, selenium and carotenoids are antioxidants that act to reduce development of reactive species that can initiate chronic disease development through inflammation. Vegetables, fruits, whole grains, legumes and nuts that contain these and other nutrients may provide anti-inflammatory benefits. Studies suggest that excessive amounts of red and processed meats, refined grains and sugar-sweetened beverages act through a variety of mechanisms to increase inflammation [49, 50].

Importantly, the impact of diet in modulating inflammation is thought to be due to complex interactions between foods and nutrients having bioactive properties [15]. Accordingly, studies have highlighted the need to characterise the relationship between diet and systemic inflammation through assessment of dietary patterns, as dietary indices consider the fact that foods are eaten in combination, thus removing the limitation that single nutrients may not reflect the overall quality of diet as a whole and are restricted in their ability to take into account interactions among nutrients [51]. Reinforcing this concept are results from the Nurses’ Health Study; Fung et. al found beneficial effects of adhering to the DASH diet in reducing inflammation in 24-year follow-up [18]. These findings are supported in a meta-analysis of randomised controlled trials which suggest that the beneficial effects of the DASH diet on reducing chronic disease risk are due not only to reductions in blood pressure, but also to improvements in inflammatory biomarker levels [52, 53]. In a study which examined the effects of the MD in men with metabolic syndrome, Richard et. al found that even in the absence of weight loss, consuming the MD significantly reduced inflammation [54]. Both the DII and E-DII scores have been validated against inflammatory biomarkers in previous research [15, 24, 51]. In addition, a recent literature review which examined 60 cross-sectional studies found an association between higher quality diet (mostly MD and anti-inflammatory diet scores) and more favourable inflammatory biomarker levels [26].

Nevertheless, it should be noted that the study by Hart et. al [26] observed that less than half the studies included in their review examined more than one inflammatory biomarker, with CRP being the most commonly assessed biomarker of systemic inflammation in studies reporting dietary score/inflammation relationships. The review found IL-6 to be the second most examined inflammatory biomarker in dietary studies. However, associations between dietary patterns and IL-6 were found to be less consistent. This inconsistency may be due to the shorter half-life of IL-6 such that CRP is likely to remain in the serum longer and because IL-6 triggers production of CRP by the liver [55]. This highlights the complex nature of inflammatory pathways when trying to assess overall inflammatory status [26].

In the current study, which compared four dietary indices with a range of biomarkers of chronic low-grade inflammation, we noted correlations between dietary quality and concentrations of C3, CRP, IL-6, TNF-α, resistin, WBC, neutrophils, the NLR and monocytes. In addition, we observed the DASH score to demonstrate a greater number of significant relationships with inflammatory biomarkers and white blood cells when compared to the DII and E-DII in linear regression models which accounted for multiple testing. This finding is interesting and unexpected as the DII is a relatively new dietary score, based on peer-reviewed research [51], which was developed specifically to provide a summary measure of diet-associated inflammation that could be used in any human population; the E-DII is a refinement of the DII which aimed to address methodological issues concerning total energy and nutrient intake and energy and nutrient densities [41]. However, our results suggest that the DASH score may be superior to both of these indices in describing the association with inflammatory biomarkers. Future modifications and improvements in dietary scores which aim to measure inflammatory status according to dietary intake may be warranted.

Also of interest is that we found the DASH score to be related to a greater number inflammatory/immunity biomarkers compared to the MD score, despite both indices emphasising consumption of fruits, vegetables, nuts and legumes. Differences in how individual dietary components within each score are weighted may account for this finding. Noticeably, we found poorer dietary quality defined by the DASH score to be more strongly correlated with consumption of red meat, sweet snacks and daily consumption of high fat/sugar foods and drinks based on food pyramid shelf servings. Previous research by our group which examined individual Healthy Eating Index-2015 dietary score components found that fatty acids, saturated fats and added sugars demonstrated significant correlations with a number of inflammatory and thrombotic biomarkers [17]. However, the MD score used in our research was constructed using a method proposed for use in non-Mediterranean countries such as Ireland [20, 38]. Different iterations of the MD score might have produced different findings. Bearing this in mind, it should be noted that a recent systematic review and meta-analysis of randomised controlled trials which compared the MD and DASH indices found that the MD appeared as the dietary pattern that showed the most prominent reduction of inflammatory biomarkers [56]. Nevertheless, studies included in this review which examined the DASH score were restricted to observing reductions in CRP levels only. Future research should examine individual dietary component associations with inflammatory and thrombotic biomarkers that demonstrated relationships with dietary scores in our study. In addition, future large-scale intervention studies are warranted to allow direct comparison of various dietary patterns in relation to a range of biomarkers reflecting multiple inflammatory and immune-related pathways [56].

### Strengths and limitations

This study has several strengths. With the elderly population growing [57] it is to be expected that the number of patients with non-communicable diseases will increase. Modifications in certain lifestyle behaviours and adopting a healthier diet may help prevent against systemic inflammation and this may be of particular importance to older adults. As far as we are aware, this research is the first to compare DASH, MD, DII and E-DII relationships with a wide range of markers of chronic low-grade inflammation and raised immune activation in a middle- to older-aged population; thus, our study has examined the greatest number of biomarkers in a relatively large population in this context. Research on dietary indices is important for public health, as studies on these can provide better insights into disease causation. Other strengths include equal representation by sex (49% male) and the use of validated questionnaires to collect data. Furthermore, to address the issue of multiple testing, we applied a stringent Romano-Wolf multiple hypothesis correction [44], which is more powerful than earlier multiple testing procedures. Nevertheless, given similar correlative strengths that were observed between dietary indices and biomarkers in non-parametric analyses, it should be noted that although correcting for multiple testing reduces the probability of false significant findings, it might also increase the probability of false negative results.

Other limitations should be noted. The cross-sectional study design, which precludes drawing conclusions regarding the temporal direction of relationships, limits inference with respect to causality. This should be considered in light of decades of work on the association between diet and serum lipids which suggest that relationships may be discernible only using longitudinal data [58]. In addition, the use of self-reported questionnaires is subject to potential inaccuracies, recall and reporting bias, including response sets such as social approval, social desirability [59–61] and residual confounding arising from imprecise measurement of variables. Another potential limitation of this study is the non-availability of information on the remaining 19 food parameters for the DII and E-DII calculations. However, on average, we have had data on 26 food parameters for DII and E-DII score calculations and previous research by our group which looked at the impact of including fewer food items to generate scores found that there was no change in relationships when going from 45 to less than 30 food parameters [24, 62]. The diet of our study population is quite similar/monotonous. Therefore, it is likely that the missing variables (which would not be frequently consumed or in large quantities) would have little to no impact on DII scores in this population. Of course, homogeneity of diet will increase the likelihood of not detecting a true relationship between diet and inflammatory markers, especially against the background of measurement imprecision [63]. Consequently, we acknowledge that where the study population and their eating habits/food culture and preferences are more diverse, then there may be a greater impact of missing items if they are ones with more anti/pro-inflammatory effects and/or are consumed in large amounts.

Finally, and related to the previous point, the generalisability of our findings may be limited. Our data were collected from a single primary care-based sample which may not be representative of the general population. However, Ireland represents a generally ethnically homogeneous population [64]. In addition, previous research suggests that approximately 98% of Irish adults are registered with a GP and that, even in the absence of a universal patient registration system, it is possible to perform population-based epidemiological studies that are representative using our methods [65].

## Conclusions

In conclusion, findings from this research demonstrate that better diet quality, determined by the DASH score, may be more closely associated with inflammatory biomarkers related to health in middle- to older-aged adults than the MD, DII and E-DII scores. Consequently, although results suggest reduced systemic inflammation as a potential biological mechanism linking a higher quality healthy diet with beneficial health effects, future modifications and improvements in these dietary indices may be warranted. Increasing understanding of the relationships between diet and markers of health is needed. This should include improved methods of measuring diet, choosing populations to study with greater dietary diversity, further examination of individual dietary components and dietary scores in relation to a wide range of biomarkers reflecting multiple inflammatory and immune-related pathways and examining longitudinal changes in both diet and markers of health. The overall goal is to inform public health planning and policy to improve and maintain optimal health at a population level.

## Supplementary Information

Below is the link to the electronic supplementary material.

Supplementary file1 (DOCX 35 KB)

Supplementary file2 (DOCX 22 KB)

## Abbreviations

BMI: Body mass index
C3: Complement component 3
CI: Confidence interval
CRP: C-reactive protein
CVD: Cardiovascular disease
DASH: Dietary approaches to stop hypertension
DII: Dietary inflammatory index
E-DII: Energy-adjusted dietary inflammatory index
FDR: False discovery rate
FFQ: Food frequency questionnaire
HbA_1c_: Glycated haemoglobin A_1c_
IL-6: Interleukin 6
IPAQ: International physical activity questionnaire
MD: Mediterranean diet
OR: Odds ratio
TNF-α: Tumour necrosis factor alpha
PAI-1: Plasminogen activator inhibitor 1
WBC: White blood cell count
NLR: Neutrophil-to-lymphocyte ratio
