# Supplementary material for: Dietary score associations with markers of chronic low-grade inflammation: a cross-sectional comparative analysis of a middle- to older-aged population
Source: Eur J Nutr. 2022 May 5;61(7):3377–90. doi: 10.1007/s00394-022-02892-1 (PMC9464136; doi:10.1007/s00394-022-02892-1)
Supplement: Supplementary file 1 — Supplementary file1 (DOCX 35 KB) [file 394_2022_2892_MOESM1_ESM.docx]

**Table S1. Descriptive characteristics and inflammatory profiles of the study population according to dietary index quartiles.**

| **Variable** | **Dietary index quartiles (n = 1862)** | | | | |
| --- | --- | --- | --- | --- | --- |
|  | Q1 | Q2 | Q3 | Q4 | *p* *_trend_* |
| **DASH score** |  |  |  |  |  |
| Male (%) | 362 (70.6) | 264 (52.1) | 196 (41.4) | 89 (24.2) | <.001 |
| Age (median) | 58.0 (54.0–63.0) | 60.0 (55.0–64.0) | 58.9 (54.0–63.0) | 59.7 (55.0–64.0) | .016 |
| Primary education only (%) | 166 (33.9) | 133 (27.4) | 102 (22.9) | 60 (17.5) | <.001 |
| On anti-inflammatory medications (%) | 77 (15.3) | 89 (17.8) | 77 (16.5) | 56 (15.6) | .974 |
| Type 2 diabetes (%) | 44 (8.6) | 52 (10.3) | 40 (8.4) | 23 (6.2) | .181 |
| CVD (%) | 54 (10.5) | 61 (12.0) | 50 (10.5) | 28 (7.6) | .157 |
| Cancer (%) | 12 (2.3) | 23 (4.5) | 18 (3.8) | 19 (5.2) | .059 |
| Never smoker (%) | 245 (48.3) | 253 (50.1) | 253 (54.1) | 206 (56.4) | <.001 |
| Former smoker (%) | 160 (31.6) | 177 (35.0) | 156 (33.3) | 130 (35.6) |  |
| Current smoker (%) | 102 (20.1) | 75 (14.9) | 59 (12.6) | 29 (7.9) |  |
| Low-level physical activity (%) | 248 (51.9) | 243 (50.3) | 204 (43.9) | 146 (41.2) | <.001 |
| BMI [kg/m^2^] (mean) | 28.6 ± 4.2 | 28.5 ± 4.8 | 28.7 ± 5.0 | 27.8 ± 4.2 | .022 |
| C3, mg/dl (mean) | 135.59 ± 25.0 | 137.00 ± 23.4 | 135.67 ± 26.1 | 133.67 ± 23.8 | .187 |
| CRP, ng/ml (median) | 1.39 (0.98–2.36) | 1.35 (0.95–2.29) | 1.39 (0.98–2.46) | 1.23 (0.94–1.88) | .019 |
| IL-6, pg/ml (median) | 1.96 (1.28–3.19) | 1.78 (1.19–2.96) | 1.75 (1.15–2.86) | 1.54 (1.11–2.29) | <.001 |
| TNF-α, pg/ml (median) | 6.22 (5.15–7.59) | 5.97 (4.91–7.34) | 5.75 (4.78–7.07) | 5.76 (4.60–7.08) | .003 |
| Adiponectin, ng/ml (median) | 3.94 (2.66–6.15) | 4.70 (2.86–7.18) | 5.08 (3.09–8.03) | 5.76 (3.75–8.95) | <.001 |
| Leptin, ng/ml (median) | 1.62 (1.00–2.78) | 1.91 (1.12–2.99) | 2.00 (1.06–3.27) | 2.09 (1.17–3.37) | .023 |
| Resistin, ng/ml (median) | 5.08 (3.86–6.77) | 4.88 (3.89–6.53) | 5.08 (4.00–6.77) | 5.05 (3.90–6.55) | .711 |
| PAI-1, ng/ml (mean) | 28.09 ± 156.8 | 27.70 ± 11.9 | 27.85 ± 13.7 | 25.02 ± 11.1 | .001 |
| WBC, 10^9^/l (median) | 5.90 (5.10–7.20) | 5.70 (4.80–6.90) | 5.60 (4.80–6.70) | 5.30 (4.40–6.10) | <.001 |
| Neutrophils, 10⁹/l (median) | 3.39 (2.71–4.28) | 3.09 (2.50–3.90) | 3.11 (2.53–3.93) | 2.77 (2.25–3.50) | <.001 |
| Lymphocytes, 10⁹/l (median) | 1.74 (1.41–2.17) | 1.81 (1.45–2.18) | 1.73 (1.42–2.11) | 1.69 (1.38–2.11) | .099 |
| NLR (median) | 1.88 (1.49–2.43) | 1.74 (1.37–2.19) | 1.81 (1.42–2.29) | 1.64 (1.26–2.13) | <.001 |
| Monocytes, 10⁹/l (median) | 0.55 (0.43–0.67) | 0.50 (0.41–0.63) | 0.49 (0.39–0.60) | 0.45 (0.36–0.56) | <.001 |
| Eosinophils, 10⁹/l (median) | 0.18 (0.12–0.26) | 0.18 (0.11–0.27) | 0.16 (0.11–0.26) | 0.16 (0.10–0.23) | .003 |
| Basophils, 10⁹/l (median) | 0.03 (0.02–0.04) | 0.03 (0.02–0.04) | 0.03 (0.02–0.04) | 0.03 (0.02–0.04) | .684 |
| **MD score** |  |  |  |  |  |
| Male (%) | 305 (45.5) | 168 (48.7) | 304 (47.4) | 134 (65.4) | <.001 |
| Age (median) | 59.0 (54.0–64.0) | 59.0 (54.5–64.0) | 59.0 (55.0–63.0) | 57.0 (53.0–63.0) | .216 |
| Primary education only (%) | 194 (30.3) | 82 (25.0) | 146 (24.3) | 39 (20.2) | .002 |
| On anti-inflammatory medications (%) | 85 (12.8) | 63 (18.6) | 112 (17.9) | 39 (19.2) | .007 |
| Type 2 diabetes (%) | 59 (8.8) | 27 (7.8) | 52 (8.1) | 21 (10.2) | .84 |
| CVD (%) | 54 (8.0) | 44 (12.8) | 69 (10.8) | 26 (12.7) | .046 |
| Cancer (%) | 28 (4.2) | 8 (2.3) | 31 (4.8) | 5 (2.4) | .846 |
| Never smoker (%) | 355 (53.1) | 194 (56.6) | 318 (50.4) | 90 (44.3) | .966 |
| Former smoker (%) | 193 (28.9) | 105 (30.6) | 237 (37.6) | 88 (43.3) |  |
| Current smoker (%) | 120 (18.0) | 44 (12.8) | 76 (12.0) | 25 (12.3) |  |
| Low-level physical activity (%) | 355 (55.6) | 149 (45.3) | 265 (43.2) | 72 (36.0) | <.001 |
| BMI [kg/m^2^] (mean) | 28.6 ± 4.7 | 28.4 ± 4.7 | 28.4 ± 4.7 | 28.2 ± 3.9 | .376 |
| C3, mg/dl (mean) | 137.35 ± 25.9 | 134.35 ± 25.5 | 135.22 ± 23.7 | 133.41 ± 21.5 | .078 |
| CRP, ng/ml (median) | 1.44 (1.00–2.44) | 1.35 (0.99–2.33) | 1.27 (0.94–2.14) | 1.19 (0.92–1.95) | .006 |
| IL-6, pg/ml (median) | 1.87 (1.24–3.00) | 1.81 (1.18–3.21) | 1.66 (1.17–2.67) | 1.74 (1.15–2.53) | .131 |
| TNF-α, pg/ml (median) | 6.13 (4.92–7.35) | 5.89 (4.93–7.30) | 5.92 (4.89–7.22) | 5.75 (4.74–7.16) | .15 |
| Adiponectin, ng/ml (median) | 4.93 (3.10–7.71) | 4.95 (2.98–7.23) | 4.69 (2.93–7.79) | 4.47 (2.58–6.22) | .216 |
| Leptin, ng/ml (median) | 1.91 (1.15–3.19) | 1.97 (1.11–3.26) | 1.92 (1.04–3.00) | 1.90 (0.63–2.76) | .977 |
| Resistin, ng/ml (median) | 5.15 (3.90–6.73) | 4.95 (3.89–6.62) | 4.98 (3.95–6.74) | 5.05 (3.87–6.54) | .673 |
| PAI-1, ng/ml (mean) | 27.36 ± 12.3 | 26.28 ± 12.3 | 27.86 ± 13.1 | 27.29 ± 11.1 | .663 |
| WBC, 10^9^/l (median) | 5.90 (4.90–7.00) | 5.70 (4.70–6.70) | 5.50 (4.70–6.50) | 5.60 (4.85–6.90) | .001 |
| Neutrophils, 10⁹/l (median) | 3.21 (2.59–4.08) | 3.13 (2.46–3.89) | 3.00 (2.46–3.76) | 3.03 (2.43–4.01) | .004 |
| Lymphocytes, 10⁹/l (median) | 1.77 (1.43–2.19) | 1.74 (1.41–2.13) | 1.70 (1.38–2.10) | 1.78 (1.51–2.17) | .269 |
| NLR (median) | 1.80 (1.43–2.30) | 1.77 (1.39–2.29) | 1.76 (1.40–2.29) | 1.76 (1.31–2.19) | .823 |
| Monocytes, 10⁹/l (median) | 0.51 (0.40–0.63) | 0.50 (0.40–0.62) | 0.48 (0.40–0.60) | 0.50 (0.40–0.63) | .034 |
| Eosinophils, 10⁹/l (median) | 0.18 (0.11–0.26) | 0.17 (0.11–0.24) | 0.17 (0.11–0.29) | 0.18 (0.11–0.29) | .561 |
| Basophils, 10⁹/l (median) | 0.03 (0.02–0.04) | 0.03 (0.02–0.04) | 0.03 (0.02–0.04) | 0.03 (0.02–0.04) | .863 |
| **DII score** |  |  |  |  |  |
| Male (%) | 180 (38.6) | 207 (44.5) | 251 (53.9) | 273 (58.7) | <.001 |
| Age (median) | 58.0 (54.0–63.0) | 59.0 (54.0–63.0) | 59.0 (54.0–64.0) | 60.0 (55.0–65.0) | .004 |
| Primary education only (%) | 86 (19.5) | 91 (20.8) | 114 (25.9) | 170 (38.3) | <.001 |
| On anti-inflammatory medications (%) | 70 (15.3) | 79 (17.3) | 79 (17.1) | 71 (15.6) | .932 |
| Type 2 diabetes (%) | 38 (8.2) | 36 (7.7) | 34 (7.3) | 51 (11.0) | .168 |
| CVD (%) | 39 (8.4) | 51 (11.0) | 56 (12.0) | 47 (10.1) | .321 |
| Cancer (%) | 16 (3.4) | 20 (4.3) | 22 (4.7) | 14 (3.0) | .833 |
| Never smoker (%) | 234 (50.9) | 249 (54.1) | 257 (55.6) | 217 (46.9) | .006 |
| Former smoker (%) | 179 (38.9) | 152 (33.0) | 138 (29.9) | 154 (33.3) |  |
| Current smoker (%) | 47 (10.2) | 59 (12.8) | 67 (14.5) | 92 (19.9) |  |
| Low-level physical activity (%) | 194 (42.9) | 200 (45.0) | 211 (48.0) | 236 (53.2) | .001 |
| BMI [kg/m^2^] (mean) | 28.1 ± 4.7 | 28.3 ± 4.5 | 28.7 ± 4.7 | 28.6 ± 4.6 | .058 |
| C3, mg/dl (mean) | 134.11 ± 24.0 | 134.56 ± 26.8 | 136.45 ± 20.6 | 137.39 ± 26.7 | .024 |
| CRP, ng/ml (median) | 1.21 (0.90–2.14) | 1.35 (0.98–2.18) | 1.37 (1.01–2.31) | 1.43 (0.96–2.45) | .037 |
| IL-6, pg/ml (median) | 1.68 (1.18–2.58) | 1.62 (1.13–2.59) | 1.88 (1.26–2.91) | 1.95 (1.23–3.18) | .024 |
| TNF-α, pg/ml (median) | 5.74 (4.70–7.14) | 5.81 (4.74–7.21) | 6.13 (5.10–7.41) | 6.09 (4.94–7.38) | .007 |
| Adiponectin, ng/ml (median) | 5.26 (3.14–7.73) | 4.95 (3.15–7.75) | 4.47 (2.70–7.35) | 4.47 (2.87–6.97) | .001 |
| Leptin, ng/ml (median) | 1.87 (1.02–3.21) | 2.00 (1.16–3.00) | 1.92 (1.10–3.05) | 1.86 (1.00–3.18) | .8 |
| Resistin, ng/ml (median) | 4.81 (3.78–6.52) | 5.05 (3.93–6.73) | 4.92 (3.92–6.71) | 5.29 (4.01–6.80) | .255 |
| PAI-1, ng/ml (mean) | 26.80 ± 14.1 | 26.73 ± 11.5 | 27.95 ± 12.1 | 27.80 ± 11.9 | .106 |
| WBC, 10^9^/l (median) | 5.40 (4.55–6.30) | 5.60 (4.70–6.70) | 5.80 (4.95–6.80) | 5.95 (5.00–7.13) | <.001 |
| Neutrophils, 10⁹/l (median) | 2.86 (2.28–3.65) | 3.04 (2.49–3.81) | 3.23 (2.55–4.00) | 3.31 (2.62–4.27) | <.001 |
| Lymphocytes, 10⁹/l (median) | 1.74 (1.44–2.12) | 1.74 (1.40–2.15) | 1.75 (1.42–2.12) | 1.76 (1.42–2.18) | .991 |
| NLR (median) | 1.63 (1.31–2.12) | 1.78 (1.35–2.28) | 1.81 (1.44–2.36) | 1.84 (1.46–2.36) | .004 |
| Monocytes, 10⁹/l (median) | 0.46 (0.37–0.57) | 0.49 (0.40–0.60) | 0.51 (0.41–0.63) | 0.53 (0.42–0.68) | <.001 |
| Eosinophils, 10⁹/l (median) | 0.16 (0.10–0.24) | 0.18 (0.12–0.27) | 0.18 (0.11–0.25) | 0.18 (0.12–0.26) | .027 |
| Basophils, 10⁹/l (median) | 0.03 (0.02–0.04) | 0.03 (0.02–0.04) | 0.03 (0.02–0.04) | 0.03 (0.02–0.04) | .427 |
| **E-DII score** |  |  |  |  |  |
| Male (%) | 153 (32.8) | 208 (44.7) | 250 (53.6) | 300 (64.5) | <.001 |
| Age (median) | 59.0 (55.0–64.0) | 59.0 (54.0–63.0) | 59.0 (55.0–64.0) | 58.0 (54.0–63.0) | .265 |
| Primary education only (%) | 85 (19.6) | 111 (25.2) | 132 (29.7) | 133 (30.0) | <.001 |
| On anti-inflammatory medications (%) | 76 (16.6) | 87 (19.0) | 73 (15.9) | 63 (13.8) | .134 |
| Type 2 diabetes (%) | 31 (6.7) | 45 (9.7) | 42 (9.0) | 41 (8.8) | .314 |
| CVD (%) | 44 (9.4) | 52 (11.2) | 54 (11.6) | 43 (9.2) | .979 |
| Cancer (%) | 19 (4.1) | 24 (5.2) | 18 (3.9) | 11 (2.4) | .108 |
| Never smoker (%) | 241 (52.3) | 241 (52.5) | 251 (54.2) | 224 (48.5) | .018 |
| Former smoker (%) | 164 (35.6) | 162 (35.3) | 155 (33.5) | 142 (30.7) |  |
| Current smoker (%) | 56 (12.1) | 56 (12.2) | 57 (12.3) | 96 (20.8) |  |
| Low-level physical activity (%) | 192 (42.4) | 194 (43.4) | 220 (49.1) | 235 (54.4) | <.001 |
| BMI [kg/m^2^] (mean) | 28.1 ± 4.6 | 28.1 ± 4.5 | 28.9 ± 4.8 | 28.6 ± 4.5 | .141 |
| C3, mg/dl (mean) | 133.73 ± 24.8 | 136.45 ± 23.6 | 136.54 ± 25.0 | 135.60 ± 25.1 | .232 |
| CRP, ng/ml (median) | 1.23 (0.93–2.07) | 1.33 (0.97–2.26) | 1.40 (0.98–2.34) | 1.43 (1.00–2.37) | .048 |
| IL-6, pg/ml (median) | 1.58 (1.15–2.59) | 1.69 (1.13–2.64) | 1.88 (1.18–3.03) | 1.96 (1.29–3.04) | <.001 |
| TNF-α, pg/ml (median) | 5.77 (4.66–7.15) | 5.74 (4.71–7.16) | 5.96 (5.02–7.22) | 6.31 (5.16–7.59) | <.001 |
| Adiponectin, ng/ml (median) | 5.37 (3.31–8.16) | 5.04 (3.22–7.95) | 4.45 (2.82–7.54) | 4.07 (2.67–6.54) | <.001 |
| Leptin, ng/ml (median) | 1.93 (1.07–3.30) | 2.00 (1.04–3.15) | 1.98 (1.09–3.25) | 1.79 (1.07–2.86) | .567 |
| Resistin, ng/ml (median) | 5.15 (3.94–6.69) | 4.78 (3.82–6.42) | 4.83 (3.85–6.52) | 5.32 (4.02–7.12) | .007 |
| PAI-1, ng/ml (mean) | 27.53 ± 14.5 | 26.48 ± 10.9 | 27.38 ± 12.2 | 27.91 ± 12.1 | .435 |
| WBC, 10^9^/l (median) | 5.50 (4.60–6.50) | 5.60 (4.75–6.55) | 5.70 (4.80–6.72) | 5.90 (5.10–7.20) | <.001 |
| Neutrophils, 10⁹/l (median) | 2.94 (2.37–3.78) | 2.99 (2.43–3.70) | 3.14 (2.52–3.97) | 3.39 (2.69–4.29) | <.001 |
| Lymphocytes, 10⁹/l (median) | 1.72 (1.43–2.12) | 1.76 (1.45–2.14) | 1.73 (1.38–2.12) | 1.76 (1.44–2.20) | .653 |
| NLR (median) | 1.72 (1.32–2.20) | 1.67 (1.35–2.18) | 1.79 (1.42–2.34) | 1.86 (1.48–2.39) | .003 |
| Monocytes, 10⁹/l (median) | 0.47 (0.38–0.60) | 0.49 (0.40–0.60) | 0.51 (0.41–0.62) | 0.53 (0.42–0.67) | .003 |
| Eosinophils, 10⁹/l (median) | 0.16 (0.11–0.24) | 0.18 (0.11–0.27) | 0.17 (0.11–0.26) | 0.17 (0.12–0.27) | .151 |
| Basophils, 10⁹/l (median) | 0.03 (0.02–0.04) | 0.03 (0.02–0.04) | 0.03 (0.02–0.04) | 0.03 (0.02–0.04) | .92 |

Abbreviations: C3: complement component 3; CRP: c-reactive protein; CVD: cardiovascular disease; DASH: Dietary Approaches to Stop Hypertension; DII: Dietary Inflammatory Index; E-DII: Energy-adjusted Dietary Inflammatory Index; IL-6: interleukin 6; MD: Mediterranean Diet; TNF-α: tumour necrosis factor-alpha; PAI-1: plasminogen activator inhibitor 1; WBC: white blood cell count; NLR: neutrophil-to-lymphocyte ratio.

Mean ± one standard deviation, median (interquartile range) and numbers (percentages) are shown. *p* for trend determined from a Jonckheere test, a linear-by-linear chi-square or an ANOVA. For the DASH and MD, lower scores represent poorer and higher scores represent better quality diet. For the DII and E-DII, higher scores are more pro-inflammatory and lower scores are anti-inflammatory.
