# Supplementary material for: Dietary score associations with markers of chronic low-grade inflammation: a cross-sectional comparative analysis of a middle- to older-aged population
Source: Eur J Nutr. 2022 May 5;61(7):3377–90. doi: 10.1007/s00394-022-02892-1 (PMC9464136; doi:10.1007/s00394-022-02892-1)
Supplement: Supplementary file 2 — Supplementary file2 (DOCX 22 KB) [file 394_2022_2892_MOESM2_ESM.docx]

**Table S2. Partial Spearman correlation coefficients between dietary scores and inflammatory and thrombotic biomarkers – excluding current smokers.**

| **Biomarker** | **DASH score** | | **MD score** | | **DII score** | | **E-DII score** | |
| --- | --- | --- | --- | --- | --- | --- | --- | --- |
|  | ρ coefficient | *p* | ρ coefficient | *p* | ρ coefficient | *p* | ρ coefficient | *p* |
| C3, mg/dl | -0.083 | **.002** | -0.028 | .297 | 0.056 | **.039** | 0.053 | .051 |
| CRP, ng/ml | -0.064 | **.018** | -0.072 | **.007** | 0.070 | **.009** | 0.077 | **.005** |
| IL-6, pg/ml | -0.070 | **.01** | -0.046 | .092 | 0.073 | **.007** | 0.096 | **<.001** |
| TNF-α, pg/ml | -0.104 | **<.001** | -0.063 | **.02** | 0.088 | **.001** | 0.103 | **<.001** |
| Adiponectin, ng/ml | -0.002 | .944 | -0.048 | .077 | 0.000 | .997 | 0.026 | .338 |
| Leptin, ng/ml | -0.018 | .506 | -0.023 | .402 | 0.047 | .079 | 0.049 | .071 |
| Resistin, ng/ml | -0.019 | .484 | 0.025 | .351 | 0.045 | .093 | 0.040 | .136 |
| PAI-1, ng/ml | -0.023 | .397 | 0.044 | .101 | -0.003 | .923 | -0.019 | .474 |
| WBC, 10^9^/l | -0.067 | **.013** | -0.010 | .713 | 0.058 | **.032** | 0.037 | .167 |
| Neutrophils, 10⁹/l | -0.085 | **.002** | -0.040 | .137 | 0.088 | **.001** | 0.064 | **.018** |
| Lymphocytes, 10⁹/l | 0.017 | .54 | 0.037 | .173 | -0.028 | .298 | -0.026 | .327 |
| NLR | -0.075 | **.005** | -0.051 | .057 | 0.087 | **.001** | 0.069 | **.011** |
| Monocytes, 10⁹/l | -0.056 | **.039** | -0.012 | .644 | 0.032 | .24 | 0.013 | .621 |
| Eosinophils, 10⁹/l | -0.035 | .192 | -0.005 | .864 | 0.001 | .961 | 0.001 | .96 |
| Basophils, 10⁹/l | -0.025 | .352 | 0.019 | .477 | -0.002 | .931 | 0.014 | .61 |

Abbreviations: C3: complement component 3; CRP: c-reactive protein; DASH: Dietary Approaches to Stop Hypertension; DII: Dietary Inflammatory Index; E-DII: Energy-adjusted Dietary Inflammatory Index; IL-6: interleukin 6; MD: Mediterranean Diet; TNF-α: tumour necrosis factor-alpha; PAI-1: plasminogen activator inhibitor 1; WBC: white blood cell count; NLR: neutrophil-to-lymphocyte ratio.

Models adjusted for sex, age, education, use of anti-inflammatory medications, type 2 diabetes, CVD, cancer, never/former smoker, physical activity, BMI and total energy intake. Models which examine the E-DII score do not adjust for total energy intake.

Values are presented as partial Spearman correlation coefficients between continuous dietary scores and inflammatory and thrombotic biomarkers among the Mitchelstown Cohort (n = 1597). Significant *p* **highlighted**.

For the DASH and MD, lower scores represent poorer and higher scores represent better quality diet. For the DII and E-DII, higher scores are more pro-inflammatory and lower scores are anti-inflammatory.
